# Congenital adrenal hypoplasia, glycerol kinase deficiency and myopathy: condition requiring prompt identification. Clinical and biochemical findings in two cases

**DOI:** 10.1186/1687-9856-2013-S1-P126

**Published:** 2013-10-03

**Authors:** Rashida Vasanwala, Fabian Yap

**Affiliations:** 1 Paediatric Endocrinology, KK Women’s & Children’s Hospital, Singapore

## 

Coincident expression of otherwise unrelated inborn errors of metabolism may occur as a result of large deletions of multiple gene, referred to as the contiguous gene deletion syndrome. We describe two male infants who presented with failure to thrive and raised urinary glycerol levels, leading diagnosis of glycerol kinase deficiency, congenital adrenal hypoplasia and myopathy.These clinical diagnoses, suggest Xp21 contiguous gene deletion syndrome.

The two male infants currently 48 months and 3 months old, presented with failure to thrive at 10 months and 1 month of age respectively. On evaluation for metabolic causes of poor weight gain, urine organic acids showed high levels of glycerol. Further results revealed pseudo-hypertriglyceridemia which raised the suspicion of glycerol kinase deficiency. Both infants were persistently hyponatremic and hyperkalemic, and the synacthen test showed a suboptimal cortisol peaks. Plasma renin was high with low aldosterone indicating mineralocorticoid defiency.The older male infant who presented at 10 months of age also had significant developmental delay with muscular weakness and markedly raised creatinine kinase.He was confirmed to have Duchene Muscular Dystrophy.

Both the infants were started on steroid and salt replacement and monogen feeds containing medium chain triglycerides. Their weight improved with normalization of sodium & potassium levels. The above spectrum of clinical & biochemical features is consistent with a diagnosis of Xp21 contiguous gene deletion syndrome. Although rare, raised urinary glycerol and serum triglycerides leads to suspicion and allows early recognition of this condition, where prompt treatment can prevent life-threatening adrenal crises.

